# Recent updates in the management of urinary incontinence

**DOI:** 10.4103/0970-1591.32063

**Published:** 2007

**Authors:** Gopal H. Badlani

**Affiliations:** Department of Urology, Wake Forest University Baptist Medical Center, Winston-Salem, NC, USA

It has been said, “Urinary incontinence is a hidden epidemic”; while it might be an overstatement, voiding dysfunctions such as overactive bladder (OAB) and urinary incontinence are highly prevalent. Its magnitude in India is undetermined, even though it is well established that it affects the quality of life. In this special issue the authors have highlighted some aspects of this entity.

Dr Lemack has comprehensively reviewed the need for testing prior to surgery. It is thought-provoking, as routine ordering of urodynamic testing is the norm in many centers. Female urology as a field is evolving in India. Discrimination based on history alone is very efficient for an experienced investigator, but less so for those delving in this field occasionally. Thorough history, with a validated questionnaire, voiding diary, some measure of quantifying urinary loss, bother and quality of life assessment, urine analysis and postvoid residual (PVR) measurement is adequate in my opinion for a pure stress urinary incontinence or initial management of OAB symptoms. Patients with failed prior surgery, mixed incontinence, unexplained large PVR and those with neuropathic history may need further workup.

In order to define this better a recent panel was established under the leadership of Dr. Nitin Kekre to establish guidelines for OAB in India. Excellent guidelines are available for urinary incontinence from the WHO consensus meeting proceedings. I have included a suggestion for understanding OAB in lower socioeconomic groups. I hope to see outcomes in the near future. Conservative therapy, by Dr. Patel, includes a variety of techniques. It is important to set goals and objectives with the patient. While conservative approaches are safe, they are unlikely to result in a cure - improvement is more likely. Thus the word treatment should be replaced by management. It is driven by the motivation of the patient and the caregiver. It is labor-intensive and poorly reimbursed.

Dr. Corocos and his colleagues address the issue of stress urinary incontinence (SUI). The treatment for SUI underwent a revolutionary change with the advent of midurethral slings. Acceptance of synthetic material was largely influenced by the introduction of these tapes. While TVT and its variants are highlighted in this article, other manufacturers such as AMS, Coloplast, Boston Scientific, Cook Urological and BARD offer competing products. It is important to learn the type of mesh used as higher complications of exposure have been reported with the use of multifilament tapes and increased retention rates with stiffer meshes. The United States Food and Drug Administration's (FDA) Manufacturer and User Facility Device Experience (MAUDE) Database has recorded self-reported incidences of major complications with the use of TVT needle. I do believe they are not specific to the TVT needle, but operator-dependent. Nevertheless potential for death, vascular, bowel injury in addition to bladder and urethral injury exist with all these devices, thus it is imperative to learn the proper use of the instruments.

As regards the use of bulking agents, the concept of coaptation leading to long-term continence has to be questioned. A plethora of agents has failed to improve upon the original results of collagen injection. The cost of repeated injections is high. With the low morbidity of midurethral slings, the indications for bulking agents in my practice are limited to a few select cases.

Our group believes in the vaginal approach to prolapse repair using mesh, based on our lab data and clinical outcomes. One recent finding from Dr. Raz's group was the under-treatment of prolapse by a urologist doing midurethral slings. If you are going to use biomaterial, it is imperative that one learns “some science” behind the material. While there are no guidelines and the literature is largely filled with case-controlled studies, the scientific knowledge is based on animal experimentation. Expert opinion derived from these, such as the recent IAUGA round table publication and a comprehensive review by Dr. Kobashi *et al* should help the reader.

Male incontinence is most commonly due to sphincteric damage post radical prostatectomy in the USA. My experience in India suggests the cause is often after surgery for benign prostate and or due to neuropathic causes. Detrusor dysfunction can coexist with sphincteric incontinence in these cases as opposed to stricture with sphincteric incontinence in the cancer group. Radiation therapy given post surgery can make matters more complicated. Artificial sphincter and male sling kits are expensive, the low-cost sling as suggested by Dr. Singla has a limited experience, setting up a need for a multicenter, prospective study of the technique with a standardized protocol to legitimize its role in male incontinence on a wide scale, otherwise only the rich can be dry!!

Myelomeningocele, recognition of high-risk patients soon after birth as defined by Dr Stuart Baur was a giant step forward in the management strategies. The definition by Dr. McGuire *et al* that Detrusor Leak Point Pressure >40 cmH_2_0 was a risk factor for upper tract deterioration improved the usefulness of urodynamics as a prognosticator. It is important to realize that the duration of pressure >40 cmH_2_0 is a key element as many have a transient rise during voiding which has no influence on upper tracts. Dr. Kapoor clearly outlines an algorithm that is easy to follow but difficult to implement if attention to detail is not adhered to.

The fistula update article reports on update, with a large emphasis on modern laparoscopic and robotic techniques. It is prudent to emphasize that these new techniques are exciting and they are largely applicable to supratrigonal or trigonal fistulas away from the orifices in nonradiated patients. Patients with obstetrical fistulas (fortunately declining incidence in India) at the bladder neck or proximal urethra, trigonal fistulas needing grafts and even some supratrigonal fistulas are better served with a vaginal approach in well trained hands at a fraction of a cost. In addition the need for anesthesia is reduced and the morbidity may be less or comparable. There is no head to head trial to answer these questions. In my view, the timing of repair and approach, based on the surgeon's skill set and experience, remain of paramount importance.

I would like to thank all the contributors, in particular my recently graduated fellow Dr. Kaytan Amrute for their help in preparing this issue. To the editors of IJU, thanks for the opportunity to put this issue together.

